# Prognostic and Predictive Value of Cadherin 11 for Patients with Gastric Cancer and Its Correlation with Tumor Microenvironment: Results from Microarray Analysis

**DOI:** 10.1155/2020/8107478

**Published:** 2020-06-26

**Authors:** Zhijun Feng, Xue Li, Zhijian Ren, Jie Feng, Xiaodong He, Chongge You

**Affiliations:** Lanzhou University Second Hospital, No. 82, Cuiyingmen, Chengguan District, Lanzhou, Gansu, China

## Abstract

Gastric cancer is a disease characterized by inflammation, and epithelial-to-mesenchymal transition (EMT) and tumor-associated macrophages (TAMs) both play a vital role in epithelial-driven malignancy. In the present study, we performed an integrated bioinformatics analysis of transcriptome data from multiple databases of gastric cancer patients and worked on a biomarker for evaluating tumor prognosis. We found that cadherin 11 (CDH11) is highly expressed not only in gastric cancer tissues but also in EMT molecular subtypes and metastatic patients. Also, we obtained evidence that CDH11 has a significant correlation with infiltrating immune cells in the tumor microenvironment (TME). Our findings reflected that CDH11 likely plays an important role in tumor immune escape and could provide a prognostic biomarker and potential therapeutic target for patients with gastric cancer.

## 1. Introduction

Gastric cancer (GC) ranks fifth in global cancer incidence and is the third most significant contributor to cancer-related mortality [1]. Nevertheless, while Epstein-Barr virus (EBV) infection was added to the list of causes of GC by The Cancer Genome Atlas (TCGA) network in 2014 [2], the role of Helicobacter pylori (H. pylori) in GC has remained unshakable [3]. During H. pylori infection, the host's defense system launches an immune response aimed at annihilating bacterium, resulting in durable inflammation in the gastric mucosa followed by a series of pathological alterations that may become cancerous. Similarly, in patients with gastritis without H. pylori infection, normal cells are repeatedly stimulated by chronic inflammation for a long time and gradually become dysfunctional with tumorigenic potential, in part via recruiting immune cells into the microenvironment [4]. All of this supports the view that GC is a disease dominated by inflammation [5]. Thus, to a certain extent, changes in TME composition, especially in the types of inflammatory infiltrating cells, might provide a better microenvironment for gastric mucosa cells to obtain the capability for carcinogenesis.

In the human body, macrophages are divided into two types commonly: (1) M1 phenotype, known as classical macrophages, which has robust antigerm and antitumor activity [6, 7], and (2) M2 phenotype, known as alternatively activated macrophages, which involves in tissue remodeling, angiogenesis, and tumor formation and progression [8, 9]. Generally, tumor-associated macrophages (TAMs), as an important regulator of the tumor immune microenvironment, are similar to M2-like phenotypes and have immunosuppressive effects and have been a hot spot in research [8, 10–12]. Several studies have reported a close relationship between the infiltration levels of macrophages and tumor progression [13–15]. In GC patients, a high level of M2 macrophages is associated obviously with the status of peritoneal dissemination, angiogenesis, immune evasion, and poor prognosis [16–19]. Ectopic expression of genes in tumor tissues can induce immune cells into the tumor microenvironment (TME), directly or indirectly, with the help of inflammatory mediators secreted by GC cells or infiltrating cells [20–23].

Cadherin 11 (CDH11) is a type II classical cadherin from the cadherin superfamily of integral membrane proteins that mediate calcium-dependent cell-cell adhesion [24]. Dysregulation of CDH11 contributes to many pathologic processes like inflammation, fibrosis, cellular migration, invasion, EMT, and carcinogenesis [25, 26]. EMT is an inflammation-driven response that plays an important role in the process of chronic inflammation, becoming cancerous. In 2019, a study based on a nontumor model revealed that there are comprehensive connections between CDH11 and the major components of cellular microenvironment such as macrophages, TGF-*β*, and myofibroblasts [27]. However, studies on the potential functions and mechanisms of CDH11 in the progression and immunology of GC are few, and the topic needs to be further expounded.

In the present study, we focused on investigating the effects of CDH11 on the prognosis and progression of GC by utilizing multiple public gene expression databases such as Oncomine (https://www.oncomine.org/resource/login.html) [28], Gene Expression Omnibus (GEO, https://www.ncbi.nlm.nih.gov/gds) [29], the Gene Expression Profiling Interactive Analysis (GEPIA, http://gepia.cancer-pku.cn/index.html) [30], Tumor Immune Estimation Resource (TIMER, https://cistrome.shinyapps.io/timer/) [31], and Kaplan-Meier Plotter (KMP, http://kmplot.com/analysis/) [32]. During the analyses, we set pancreatic and colorectal cancers as controls and discussed the relationships between CDH11 and different clinical characteristics of patients with tumors. Furthermore, we also explored the possible molecular mechanisms of CDH11 involved in gastric carcinogenesis via the construction of the protein-protein interaction (PPI) and the coexpression network on the STRING database (https://string-db.org) [33] as well as investigation of the relationships between CDH11 and the levels of infiltrating cells in TME based on the TIMER database. The findings of this report reflected the important role of CDH11 in GC and revealed an underlying interaction between CDH11 and tumor immune response.

## 2. Materials and Methods

### 2.1. Gene Expression Analyses

Gene expression analyses were completed as follows: Using the Oncomine, TIMER, and GEPIA databases, we evaluated the differences in CDH11 expression between tumor and normal tissues in multiple cancer types besides gastric, pancreatic, and colorectal cancer. To exclude the impact of various annotation platforms on gene expression, we retrieved the datasets of gastric, pancreatic, and colorectal cancer annotated by the “GPL570” platform from the GEO database and then plotted the results of expression analyses into boxplots through the ggplot2 package in R software.

### 2.2. Prognostic Analysis

Survival analyses were performed using the GEPIA, GEO, and KMP databases to accurately assess the impact of CDH11 on the overall survival (OS) and disease-free survival (DFS) of patients with GC. From GEPIA, we obtained the OS data of multiple cancers, and from GEO, we downloaded only GC datasets and carried out survival analyses using survival package in R software. From KMP, not only did we obtain the OS status of all GC patients but also implemented subgroup analyses to find more evidence for the ability of CDH11 to predict the prognosis of GC. Finally, we explored the relationship between CDH11 and DFS of gastric cancer patients. The indexes of the survival analyses contained survival curves, the HR with 95% confidence intervals (95% CI), and log-rank *P* value.

### 2.3. Clinical Correlation Analyses

To better understand the potential role of CDH11 in gastric tumorigenesis, we investigated the relationships between CDH11 and various TNM stages, pathological types, T stages, molecular subtypes, and metastatic status in GC patients from the TCGA and GEO databases. Statistical tests were used to assess the differences between groups, and the results were represented by scattering and boxplots using GraphPad Prism software (https://www.graphpad.com, Version 7.0).

### 2.4. CDH11 Molecular Interaction Analysis

We constructed the PPI and the coexpression networks of CDH11 on the STRING database to explain further the potential molecular mechanisms of CDH11 involved in stomach carcinogenesis. For the PPI network, we defined the meaning of network edges as “evidence,” the active interaction sources as “textmining, experiments, and databases,” and the minimum required interaction score as “highest confidence (0.900).” For the coexpression network, the meaning of network edges was defined as “confidence,” and the minimum required interaction score as “medium confidence (0.400).” We then performed an analysis of the KEGG pathway enrichment for those molecules in the PPI network through the Enrichr database (http://amp.pharm.mssm.edu/Enrichr/) [34], and the results were visualized by the GOplot package [35].

### 2.5. TIMER Database Analysis

TIMER is a comprehensive database based on TCGA, which is dedicated to evaluating the levels of infiltrating immune cells in TME [36]. First, we recorded the effects of different immune cells on the OS of GC patients, and then, based on the strength of tumor purity, we explored the correlations among CDH11, types of infiltrating immune cells, cytokines associated with immune cells, and gene markers on the TIMER database. The cytokines and gene markers mainly involved TAMs and M1 and M2 macrophages and have been referenced in prior studies [37]. Finally, in GC patients, we generated expression scatter plots and carried out Spearman's correlation analyses via setting the *x*-axis with gene markers and the *y*-axis with CDH11 expression.

### 2.6. Statistical Analysis

GraphPad Prism is used for statistical analysis. The D'Agostino-Pearson normality test was used to describe the distribution of gene expression. An *F*-test was used to evaluate the homogeneity of variance. Student's *t*-test, one-way ANOVA, and the Mann-Whitney-Wilcoxon test were used to reveal the statistical significance between groups according to data distribution and numbers of compared groups. Kaplan-Meier analysis and log-rank test were applied to determine the survival curves. Correlations between CDH11, infiltrating cell types, and gene markers of immune cells were established by Spearman's correlation. The strength of the correlations was determined using the following guide for the absolute value: 0.00–0.10 “negligible,” 0.10–0.39 “weak,” 0.40–0.69 “moderate,” 0.70–0.89 “strong,” and 0.90–1.0 “very strong” [38]. The results were considered to have statistical significance when *P* < 0.05. Survival curves were obtained from the GEPIA sever and survival package in R software and displayed with HR and *P* value from a log-rank test.

## 3. Results

### 3.1. The Expression Levels of CDH11 in Various Human Cancers

**Figure 1:**
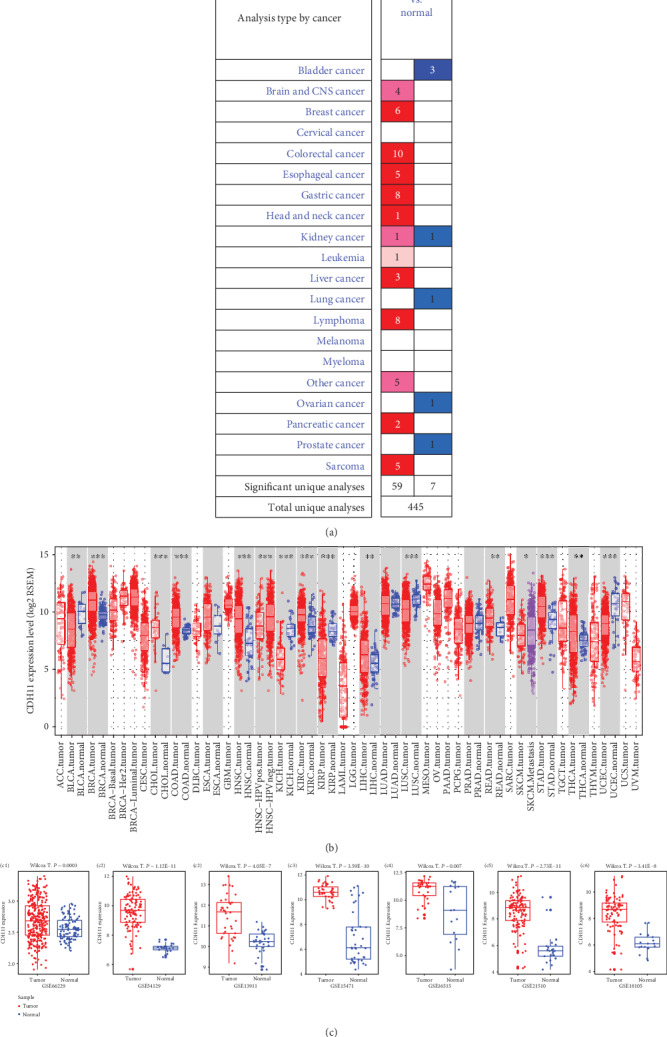
Expression levels of CDH11 in various human cancers. (a) Increased or decreased CDH11 in different cancers compared with normal tissues in the Oncomine database. (b) Expression levels of CDH11 in different tumor types from the TIMER database (^∗^*P* < 0.05, ^∗∗^*P* < 0.01, and ^∗∗∗^*P* < 0.001). (c) (c1–c3) Expression levels of CDH11 in gastric cancer datasets from the GEO database (Wilcox T.: *Wilcox*. test; *E*: exponent). (c4, c5) Expression levels of CDH11 in pancreatic cancer datasets from the GEO database. (c6, c7) Expression levels of CDH11 in colorectal cancer datasets from the GEO database. Red represents high expression levels, and blue represents low expression levels.

With Oncomine, compared with normal tissues, a high level of CDH11 expression was observed in breast, colorectal, esophageal, gastric, liver, lymphoma, pancreatic cancer, and sarcoma tissues. However, lower expressions were found in bladder, kidney, lung, ovarian, and prostate cancer tissues (Figure 1(a)). The details of CDH11 expression in gastric, colorectal, and pancreatic cancers are listed in Table S1. Data obtained from TIMER and GEPIA showed similar results, with high levels of CDH11 expression shown to be more common in adenocarcinoma like breast, gastric, pancreatic, and colorectal cancers, while lower expression mainly existed in tumors of the urinary and respiratory systems (Figure 1(b) and Figure S1). In GEO, we compared CDH11 expression between normal and cancerous tissues from the matrixes of the GSE66229 [39], GSE54129, GSE13911 [40], GSE15471 [41], GSE16515 [42], GSE21510 [43], and GSE18105 [44] datasets. The results reflected that the expression levels of CDH11 are higher in cancerous tissues than in normal tissues (Figure 1(c)), and statistical significance existed (*P* < 0.05). The corresponding information of these candidate datasets in this study is provided as Table S2.

### 3.2. Prognostic Potential of CDH11 in Cancers

**Figure 2:**
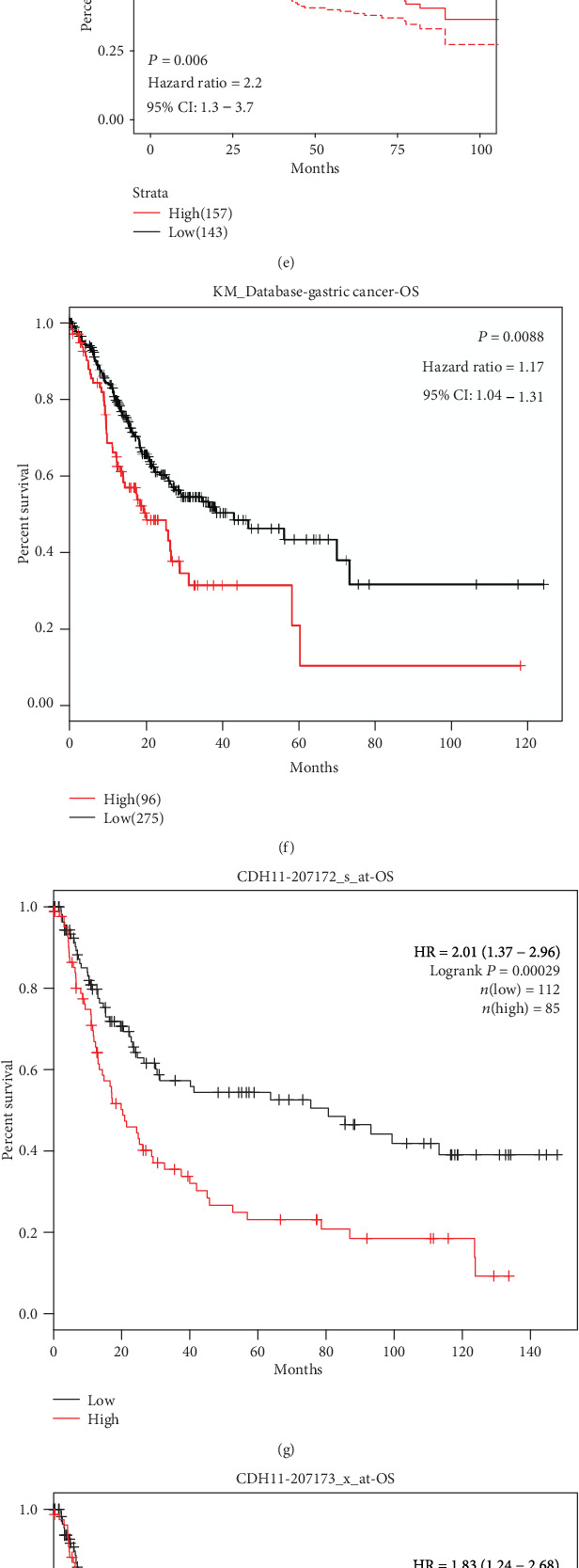
Kaplan-Meier survival curves comparing the high and low expressions of CDH11 in various cancers. (a–c) Survival curves of overall survival (OS) in STAD (stomach adenocarcinoma), PAAD (pancreatic adenocarcinoma), and COAD (colon adenocarcinoma) from the GEPIA database. (d, e) Survival curves of OS in two gastric cancer cohorts from GEO. (f) High CDH11 expression was correlated with poor OS in the RNA-seq data of GC from the KMP database. (g–i) Survival curves of high and low CDH11 expressions with different affymetrix IDs in the gene chip data of GC from the KMP database. HR: hazard ratio.

**Figure 3:**
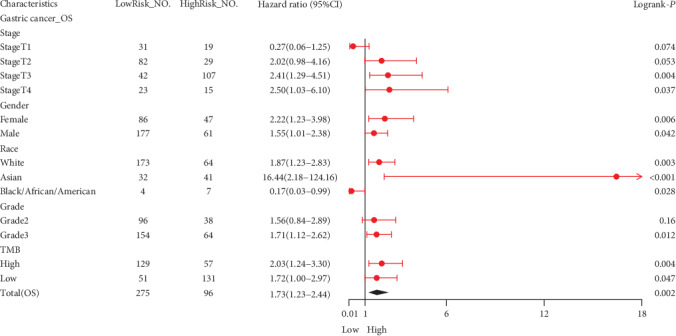
Forest plot comparing the high and low expressions of CDH11 in various clinical features on the KMP database. OS: overall survival; NO.: the number of patients with gastric cancer; 95% CI: 95% confidence interval; T1: the depth of tumor invasion arrives in submucosa; T2: the depth of tumor invasion arrives in muscularis propria; T3: the depth of tumor invasion arrives in subserosa; T4: the depth of tumor invasion arrives in serosa and/or the adjacent structure; TMB: tumor mutation burden.

In GEPIA, we confirmed that the OS of stomach adenocarcinoma (STAD) patients with a high level of CDH11 expression compared with the low-level group had statistical significance (*P* < 0.05, Figure 2(a)), but there were no significant relationships with PAAD, COAD (Figures 2(b) and 2(c)), and other types of cancers (Figure S2). Based on the results from two cohorts (GSE26253 and GSE62254), a total of 732 samples with different stages of GC, downloaded from GEO, showed that high CDH11 expression is strongly associated with poor prognosis (OS: HR = 1.20, 95%CI = 1.1 to 1.4, log-rank: *P* = 0.002 and OS: HR = 2.20, 95%CI = 1.3 to 3.7, log-rank: *P* = 0.006, respectively) (Figures 2(d) and 2(e)). Survival analysis of all GC patients using the KMP database found similar results (Figures 2(f)–2(i)). Subgroup analysis revealed that CDH11 overexpression is dramatically related to the poor prognosis of GC patients who are at T3 and T4 stages or grade 3 or have a high tumor mutation burden (TMB) (Figure 3). Also, we found evidences that CDH11 might promote the gastric cancer progression (Table S3). These results suggest that the expression level of CDH11 has a remarkable impact on the survival of GC, and CDH11 may be a good marker for predicting the prognosis of GC patients, particularly in advanced cases.

### 3.3. High CDH11 Expression Impacts the Progression of GC

**Figure 4:**
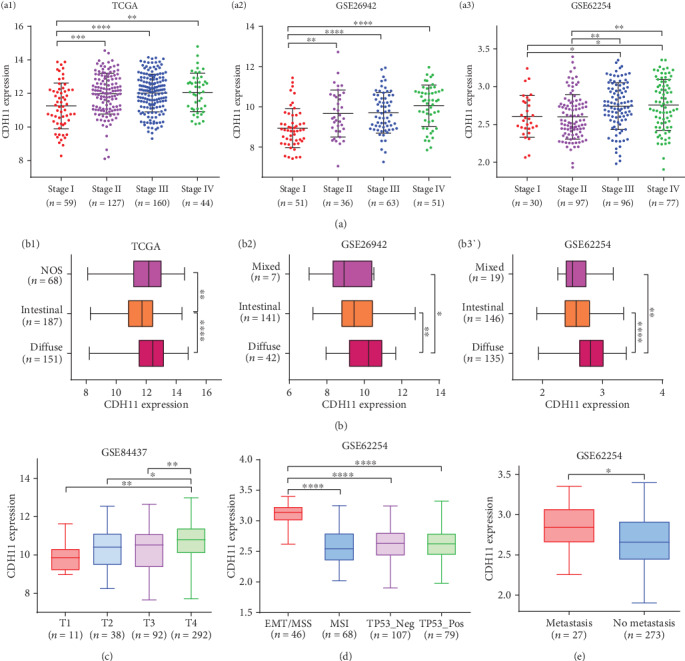
Different levels of CDH11 expression between various clinical characteristics of GC patients. (a) CDH11 expression in different AJCC stages I-IV of patients with gastric cancer (a1, analyzing STAD (stomach adenocarcinoma) data from TCGA (The Cancer Genome Atlas); a2 and a3, two gastric cancer cohorts (GSE26942 and GSE62254) from the GEO database). (b) CDH11 expression in different Lauren types of patients with gastric cancer (b1, analyzing STAD data from the TCGA database; b2 and b3, two gastric cancer cohorts (GSE26942 and GSE62254) from the GEO database). (c) CDH11 expression in different T stages of gastric cancer from GSE84437 in the GEO database. (d) CDH11 expression in different molecular types of gastric cancer from GSE62254. (e) CDH11 expression in different metastatic status of gastric cancer from GSE62254. Stages I-IV: the staging form of gastric cancer from the American Joint Committee on Cancer (AJCC); NOS: not otherwise specified; T1: the depth of tumor invasion arrives in submucosa; T2: the depth of tumor invasion arrives in muscularis propria; T3: the depth of tumor invasion arrives in subserosa; T4: the depth of tumor invasion arrives in serosa and/or the adjacent structure; EMT: epithelial-mesenchymal transition; MSS: microsatellite stability; MSI: microsatellite instability; TP53: tumor protein p53; Neg: negative; Pos: positive. ^∗^*P* < 0.05, ^∗∗^*P* < 0.01, ^∗∗∗^*P* < 0.001, and ^∗∗∗∗^*P* < 0.0001.

High levels of CDH11 expression were observed in AJCC stage IV and diffuse type of GC in TCGA (*P* < 0.05, Figures 4(a), a1 and 4(b), b1), GSE26942 [39] (*P* < 0.05, Figures 4(a), a2 and 4(b), b2), and GSE62254 [45] (*P* < 0.05, Figures 4(a), a3 and 4(b), b3). GSE84437, which included 433 GC samples, was used to evaluate the condition of CDH11 expression among different T1-T4 stages of GC, and the final results showed that the highest level of expression was in the T4 stage (*P* < 0.05, Figure 4(c)). Here, the T1-4 categories refer to the depth of tumor invasion in the submucosa, muscularis propria, subserosa, serosa, and/or the adjacent structure, respectively [46]. Apart from these, we also detected statistical differences in CDH11 expression between different molecular subtypes as well as different metastatic status in GSE62254 (*P* < 0.05, Figures 4(d) and 4(e), respectively). However, we did not find significant differences of CDH11 expression in GC patients with lymph node metastasis (Figure S3). These findings show that CDH11 contributes to the progression of GC, which thereby warrant further investigation.

### 3.4. CDH11 Molecular Interaction Analysis

**Figure 5:**
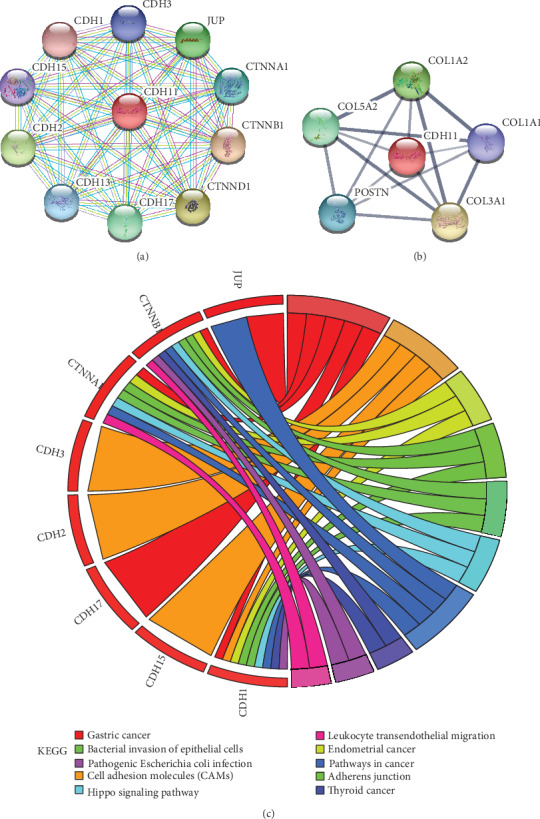
The interacting molecules of CDH11 and the KEGG pathway enrichment for interacting molecules. (a) The PPI network of CDH11 constructed on the STRING database. Purple line indicates that the source of active interaction comes from experiments, green line indicates that the source of active interaction comes from textmining, and blue and lilac lines indicate that the source of active interaction comes from databases like Biocarta, BioCyc, KEGG, and Reactome. (b) The coexpression network of CDH11 constructed on the STRING database. (c) The KEGG pathway enrichment for molecules of the PPI network from the Enrichr database. Circles represent genes, lines represent interactions between gene-encoded proteins, and line color and width present evidence of interactions between proteins.

Most CDH11-interacting molecules are members of the cadherin superfamily, according to the PPI network (Figure 5(a)). However, coexpression molecules are mainly related to the extracellular matrix (ECM), such as the collagen family and periostin (POSTN) (Figure 5(b)). The KEGG pathways of interacting molecules are enriched in carcinogenic pathways such as gastric, endometrial, and thyroid cancers. Notably, we found that leukocyte transendothelial migration, a pathway involving the TME, appears in these enriched pathways (Figure 5(c)). This evidence indicates that CDH11 may participate in ECM remodeling and the formation of the tumor immune microenvironment.

### 3.5. Relationship between CDH11 and Immune Infiltration Level in GC

**Figure 6:**
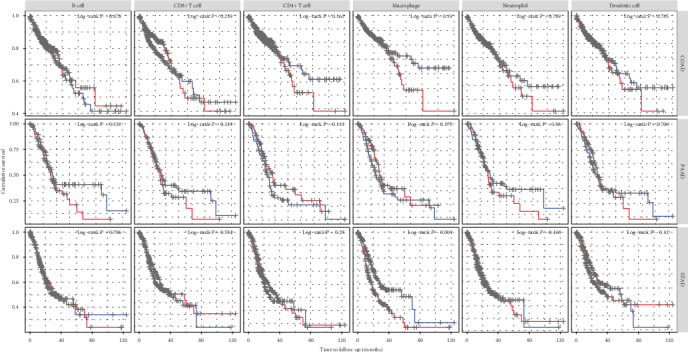
Kaplan-Meier survival curves comparing various immune cells in the tumor microenvironment (TME) of COAD (colon adenocarcinoma), PAAD (pancreatic adenocarcinoma), and STAD (stomach adenocarcinoma).

**Figure 7:**
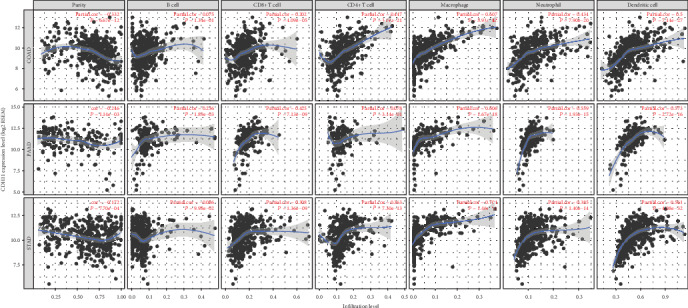
Correlation of CDH11 expression with immune infiltration level in COAD (colon adenocarcinoma), PAAD (pancreatic adenocarcinoma), and STAD (stomach adenocarcinoma).

**Figure 8:**
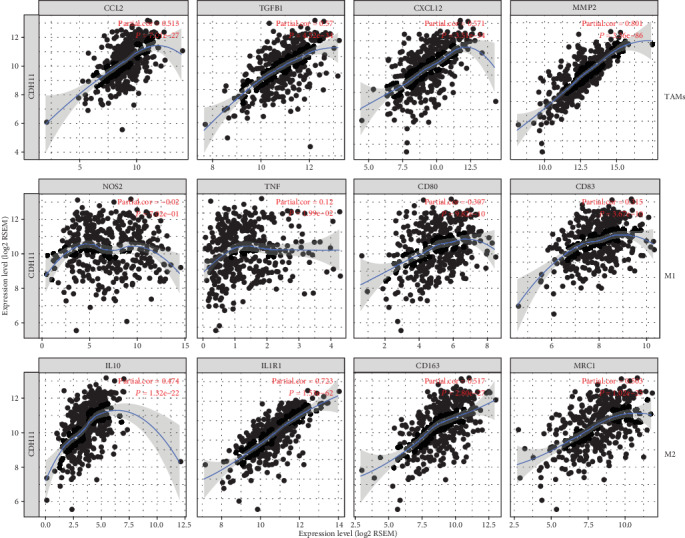
CDH11 expression correlated with macrophage polarization in STAD (stomach adenocarcinoma). Cytokines and markers include CCL2, TGFB1, CXCL12, and MMP2 of tumor-associated macrophages (TAMs); NOS2, TNF, CD80, and CD83 of M1 macrophages; and IL10, IL1R1, CD163, and MRC1 of M2 macrophages.

In the TIMER database, we found that the high infiltration level of macrophages in TME is significantly correlated with the poor prognosis of STAD patients (*P* < 0.05, Figure 6). Further analysis showed CDH11 overexpression to be strongly related to the infiltration level of macrophages in STAD (COAD: *r* = 0.607, *P* < 0.05; PAAD: *r* = 0.606, *P* < 0.05; STAD: *r* = 0.704, *P* < 0.05; Figure 7). Based on tumor purity, we ascertained that most cytokines are secreted by TAMs and M2 macrophages, and marker sets have significant correlations with CDH11 in STAD, with examples including CCL-2, TGFB1, CXCL12, and MPP2 of TAMs and IL-10, IL1R1, CD163, and MRC1 of M2 phenotype (*P* < 0.0001; Figure 8). However, NOS2, TNF, CD80, and CD83 of the M1 phenotype were shown to have a negative or weak correlation (Figure 8). Similar results were found in the GEPIA database (Table 1). These results reflect that CDH11 plays a specific role in immune infiltration of GC, especially via macrophages.

## 4. Discussion

CDH11 has been reported to play a dual role in the occurrence and development of various types of cancer. In breast cancer, CDH11 enhances the ability of cancer cells to metastasize and invade [47], while blocking it inhibits the process of EMT phenotype [48]. However, in malignant tumors of the head and neck, CDH11 is a tumor suppressor controlling the proliferation and invasion of cancer cells [25]. In GC, it was reported that CDH11 is associated with tumor progression and prognosis via regulating adhesion-related pathways [49]. Here, we reported that CDH11 not only promotes the biological process of EMT but also is closely related to a poor prognosis in GC patients, especially in those with more severe tumor stages. Furthermore, our analysis shows that there is a significant correlation between CDH11 and the infiltration levels of macrophages in the TME of GC. Thus, these findings provide a new viewpoint in realizing the potential role of CDH11 in tumor progression and immunology and its use as a cancer biomarker to predict prognosis in GC.

In the present study, we mainly discussed the value and significance of CDH11 in patients with GC via integrated bioinformatics analysis. Although the advent of high-throughput DNA sequencing has provided us with an effective tool to study the molecular pathology of tumors, different platforms often produce different sequencing results [50]. During our study, we limited the platform as “GPL570” only when analyzing the data from the GEO database to eliminate the differences from various sequencing platforms. Furthermore, tumor purity is an important confounder in evaluating the correlation between gene expression and clinicopathologic features [51]. Thus, we also accounted for the interference of tumor purity when analyzing the relationship between CDH11 and immune cell infiltrations in TME. All of these measures guaranteed the reliability of our results. Through a comprehensive analysis of CDH11 expression profiles of GC in multiple databases, we found that CDH11 is highly expressed in tumor tissues, which indicates that CDH11 may promote oncogenesis in the stomach. The results between CDH11 and the clinical features of GC patients showed that CDH11 overexpression is distinctly associated with worse pathological features such as EMT, metastatic status, higher T stage, and tumor mutation burden (TMB). Survival analysis also confirmed that a higher level of CDH11 expression has a higher HR of OS in GC patients. Similar results have been reported in previously published studies [52, 53]. Cancer is the result of a multigene and multistep process. Therefore, we constructed the PPI and the coexpression molecule networks of CDH11, and the results indicated that CDH11 might take part in matrix degradation, which is one of the significant characteristics in the process of invasion and metastasis in GC.

Another important aspect of our results is that CDH11 was found to be strongly linked to the infiltration level of diverse immune cells in different types of cancer, especially GC. CDH11 have a positive relationship not only with the infiltration level of macrophages in the TME of GC but also with cytokines secreted by macrophages and gene markers such as CCL2, CXCL12, and TGFB1 of TAMs and IL10, IL1R1, CD163, and MRC1 of M2. As inducers, CCL2, TGFB1, CXCL12, and MMP2 not only recruit more macrophages into the TME but also facilitate their polarization and the generation of more M2 macrophages [12, 54–58]. High expression of M2-related markers often reflects the increased proportion of M2 macrophages in the TME [20, 37, 59]. These findings reflect the potential capability of CDH11 to induce macrophages into the TME and accelerate the transformation of M1 to M2 by interacting with cytokines and finally regulate the formation of the immune microenvironment.

With the results of our research combined with those of previously published studies, the role of CDH11 in the development of GC may be explained by several possible mechanisms. For one, CDH11 has a positive correlation with the expression of TGFB1 encoding the protein of transforming growth factor-*β* (TGF-*β*). As we know, TGF-*β* is recognized as a powerful inducer of EMT, which is a vital step in the tumor transformation cascade [60, 61]. In the PPI network, we found that CDH11 interacts extensively with the members of the cadherin family, such as CDH1, CDH2, CDH3, CDH11, and CDH17. Among these molecules, CDH1 and CDH2 are the general markers to evaluate EMT status [62]. In addition, our analysis reveals that CDH11 is coexpressed with molecules involved in the extracellular matrix, such as COL1A2, COL1A1, COL3A1, COL5A2, and POSTN. Meanwhile, CDH11 also shows a robust positive correlation with MMP2, which is one of the notable molecules in the MMP family involved in cell adhesion, angiogenesis, and tumor progression [63–65]. Aberrant expression of matrix-related genes often leads to changes in the stroma structure and makes it easier to degrade, finally resulting in ECM remodeling, which is essential in the initiation and progression of EMT [66]. Also, CDH11 has a close relationship with the infiltration level of macrophage-related inflammatory factors (e.g., IL10, CCL2, and CXCL12). This may be the result of interaction between tumor cells with high expression of CDH11 and infiltrating immune cells in the TME. As previously mentioned, tumor-related immune cells can kill tumorous cells or promote them to progress and metastasize. Unfortunately, in most cases, immune cells in the TME become an accomplice of tumors. On the one hand, they introduce more immune cells into the microenvironment through the inflammatory mediators they or the tumor cell secreted; on the other hand, they arouse significant changes in the composition of immune cells in the TME, immunocytes with killer function decrease, immunocytes with inhibitive function increase, and an immunosuppressive microenvironment more suitable for tumor cells is eventually formed. In our study, according to the close relationship between CDH11 and macrophages, especially the M2 phenotype, we can infer that CDH11 contributes to helping cancer cells escape the immune response. Nevertheless, many unanswered issues are deserving of further investigation. In particular, how CDH11 affects cadherin family members, or which one has the biggest influence on tumorigenesis, and the exact relationships among CDH11, EMT, inflammatory cytokines, and TAMs need to be confirmed by both in vitro and in vivo experiments.

## 5. Conclusion

CDH11 participates in the biological process of EMT and induces the formation of TAMs in the TME, thus promoting the occurrence and development of GC and ultimately leading to a poor prognosis. Therefore, CDH11 likely plays a vital role in tumor immune escape and could provide a prognostic biomarker and potential therapeutic target for patients with GC.

## Figures and Tables

**Table 1:** Correlation analysis of CDH11 expression and the gene markers of macrophages in the GEPIA database.

Description	Gene marker	CDH11 in STAD			
		Normal		Tumor	
		*R*	*P*	*R*	*P*
TAMs	CCL2	0.41	∗	0.54	∗∗∗∗
	TGFB1	0.03	ns	0.65	∗∗∗∗
	CXCL12	0.74	∗∗∗∗	0.61	∗∗∗∗
	MMP2	0.75	∗∗∗∗	0.78	∗∗∗∗
M1 phenotype	NOS2	0.20	ns	0.05	ns
	TNF	-0.18	ns	0.19	∗∗∗
	CD80	-0.05	ns	0.44	∗∗∗∗
	CD83	0.40	∗	0.42	∗∗∗∗
M2 phenotype	IL10	0.30	ns	0.57	∗∗∗∗
	IL1R1	0.36	ns	0.74	∗∗∗∗
	CD163	0.58	∗∗∗	0.54	∗∗∗∗
	MRC1	0.77	∗∗∗∗	0.61	∗∗∗∗

STAD: stomach adenocarcinoma; *R*: Spearman correlation coefficient; *P*: *P* values of partial correlation analysis; TAMs: tumor-associated macrophages. ^∗^*P* < 0.05; ^∗∗^*P* < 0.01; ^∗∗∗^*P* < 0.001; ^∗∗∗∗^*P* < 0.0001. ns: no significance.

## Data Availability

The data used in the article are from public gene expression databases, such as TCGA, GEO, GEPIA, TIMER, and Oncomine.

## Supplementary Materials

Supplementary Materials Figure S1: expression levels of CDH11 in various human cancers from the GEPIA database. Figure S2: Kaplan-Meier survival curves comparing the high and low expressions of CDH11 in various cancers from the GEPIA database. Figure S3: different levels of CDH11 expression between different lymph node metastases of GC patients. Table S1: CDH11 expression in gastric, colorectal, and pancreatic cancers from the Oncomine database. Table S2: the information of datasets used for differential analysis in the study. Table S3: the relationship between CDH11 and disease progression in patients with gastric cancer.

## References

[1] Bray F., Ferlay J., Soerjomataram I., Siegel R. L., Torre L. A., Jemal A. (2018). Global cancer statistics 2018: GLOBOCAN estimates of incidence and mortality worldwide for 36 cancers in 185 countries. *CA: a Cancer Journal for Clinicians*.

[2] The Cancer Genome Atlas Research Network (2014). Comprehensive molecular characterization of gastric adenocarcinoma. *Nature*.

[3] Plummer M., Franceschi S., Vignat J., Forman D., de Martel C. (2015). Global burden of gastric cancer attributable to Helicobacter pylori. *International Journal of Cancer*.

[4] Lee K., Hwang H., Nam K. T. (2014). Immune response and the tumor microenvironment: how they communicate to regulate gastric cancer. *Gut and liver*.

[5] Nagtegaal I. D., Odze R. D., Klimstra D. (2019). The 2019 WHO classification of tumours of the digestive system. *Histopathology*.

[6] Wang N., Liang H., Zen K. (2014). Molecular mechanisms that influence the macrophage M1–M2 polarization balance. *Frontiers in Immunology*.

[7] Bashir S., Sharma Y., Elahi A., Khan F. (2016). Macrophage polarization: the link between inflammation and related diseases. *Inflammation research*.

[8] Qian B. Z., Pollard J. W. (2010). Macrophage diversity enhances tumor progression and metastasis. *Cell*.

[9] Mantovani A., Biswas S. K., Galdiero M. R., Sica A., Locati M. (2013). Macrophage plasticity and polarization in tissue repair and remodelling. *The Journal of Pathology*.

[10] Chanmee T., Ontong P., Konno K., Itano N. (2014). Tumor-associated macrophages as major players in the tumor microenvironment. *Cancers*.

[11] Shapouri-Moghaddam A., Mohammadian S., Vazini H. (2018). Macrophage plasticity, polarization, and function in health and disease. *Journal of Cellular Physiology*.

[12] Belgiovine C., D’Incalci M., Allavena P., Frapolli R. (2016). Tumor-associated macrophages and anti-tumor therapies: complex links. *Cellular and Molecular Life Sciences*.

[13] Xue Y., Tong L., LiuAnwei Liu F. (2019). Tumor‑infiltrating M2 macrophages driven by specific genomic alterations are associated with prognosis in bladder cancer. *Oncology Reports*.

[14] Cortese N., Soldani C., Franceschini B. (2019). Macrophages in colorectal cancer liver metastases. *Cancers*.

[15] Chen Y., Zhang S., Wang Q., Zhang X. (2017). Tumor-recruited M2 macrophages promote gastric and breast cancer metastasis via M2 macrophage-secreted CHI3L1 protein. *Journal of Hematology & Oncology*.

[16] Yamaguchi T., Fushida S., Yamamoto Y. (2016). Tumor-associated macrophages of the M2 phenotype contribute to progression in gastric cancer with peritoneal dissemination. *Gastric Cancer*.

[17] Zhang H., Wang X., Shen Z., Xu J., Qin J., Sun Y. (2015). Infiltration of diametrically polarized macrophages predicts overall survival of patients with gastric cancer after surgical resection. *Gastric Cancer*.

[18] Park J. Y., Sung J. Y., Lee J. (2016). Polarized CD163+ tumor-associated macrophages are associated with increased angiogenesis and CXCL12 expression in gastric cancer. *Clinics and Research in Hepatology and Gastroenterology*.

[19] Lin C., He H., Liu H. (2019). Tumour-associated macrophages-derived CXCL8 determines immune evasion through autonomous PD-L1 expression in gastric cancer. *Gut*.

[20] Chen L., Shi Y., Zhu X. (2019). IL‑10 secreted by cancer‑associated macrophages regulates proliferation and invasion in gastric cancer cells via c‑Met/STAT3 signaling. *Oncology Reports*.

[21] Zhou Z., Xia G., Xiang Z. (2019). A C-X-C chemokine receptor type 2-dominated cross-talk between tumor cells and macrophages drives gastric cancer metastasis. *Clinical Cancer Research*.

[22] Gebremariam H. G., Qazi K. R., Somiah T. (2019). Lactobacillus gasseri suppresses the production of proinflammatory cytokines in Helicobacter pylori-infected macrophages by inhibiting the expression of ADAM17. *Frontiers in Immunology*.

[23] Xu J., Yu Y., He X. (2019). Tumor-associated macrophages induce invasion and poor prognosis in human gastric cancer in a cyclooxygenase-2/MMP9-dependent manner. *American Journal of Translational Research*.

[24] Alimperti S., Andreadis S. T. (2015). CDH2 and CDH11 act as regulators of stem cell fate decisions. *Stem Cell Research*.

[25] Piao S., Inglehart R. C., Scanlon C. S., Russo N., Banerjee R., D'Silva N. J. (2017). CDH11 inhibits proliferation and invasion in head and neck cancer. *Journal of Oral Pathology & Medicine*.

[26] Row S., Liu Y., Alimperti S., Agarwal S. K., Andreadis S. T. (2016). Cadherin-11 is a novel regulator of extracellular matrix synthesis and tissue mechanics. *Journal of Cell Science*.

[27] Lodyga M., Cambridge E., Karvonen H. M. (2019). Cadherin-11-mediated adhesion of macrophages to myofibroblasts establishes a profibrotic niche of active TGF-*β*. *Science Signaling*.

[28] Rhodes D. R., Yu J., Shanker K. (2004). *ONCOMINE*: A Cancer Microarray Database and Integrated Data-Mining Platform. *Neoplasia*.

[29] Clough E., Barrett T. (2016). The Gene Expression Omnibus database. *Methods in Molecular Biology*.

[30] Tang Z., Li C., Kang B., Gao G., Li C., Zhang Z. (2017). GEPIA: a web server for cancer and normal gene expression profiling and interactive analyses. *Nucleic Acids Research*.

[31] Li B., Severson E., Pignon J. C. (2016). Comprehensive analyses of tumor immunity: implications for cancer immunotherapy. *Genome Biology*.

[32] Nagy A., Lanczky A., Menyhart O., Gyorffy B. (2018). Validation of miRNA prognostic power in hepatocellular carcinoma using expression data of independent datasets. *Scientific Reports*.

[33] Szklarczyk D., Gable A. L., Lyon D. (2019). STRING v11: protein-protein association networks with increased coverage, supporting functional discovery in genome-wide experimental datasets. *Nucleic Acids Research*.

[34] Chen E. Y., Tan C. M., Kou Y. (2013). Enrichr: interactive and collaborative HTML5 gene list enrichment analysis tool. *BMC Bioinformatics*.

[35] Walter W., Sanchez-Cabo F., Ricote M. (2015). GOplot: an R package for visually combining expression data with functional analysis: Fig. 1. *Bioinformatics*.

[36] Li T., Fan J., Wang B. (2017). TIMER: a web server for comprehensive analysis of tumor-infiltrating immune cells. *Cancer Research*.

[37] Chávez-Galán L., Olleros M. L., Vesin D., Garcia I. (2015). Much more than M1 and M2 macrophages, there are also CD169(+) and TCR(+) macrophages. *Frontiers in Immunology*.

[38] Schober P., Boer C., Schwarte L. A. (2018). Correlation coefficients. *Anesthesia & Analgesia*.

[39] Oh S. C., Sohn B. H., Cheong J. H. (2018). Clinical and genomic landscape of gastric cancer with a mesenchymal phenotype. *Nature Communications*.

[40] D’Errico M., de Rinaldis E., Blasi M. F. (2009). Genome-wide expression profile of sporadic gastric cancers with microsatellite instability. *European journal of cancer*.

[41] Idichi T., Seki N., Kurahara H. (2017). Regulation of actin-binding protein ANLN by antitumormiR-217inhibits cancer cell aggressiveness in pancreatic ductal adenocarcinoma. *Oncotarget*.

[42] Pei H., Li L., Fridley B. L. (2009). FKBP51 affects cancer cell response to chemotherapy by negatively regulating Akt. *Cancer Cell*.

[43] Tsukamoto S., Ishikawa T., Iida S. (2011). Clinical significance of osteoprotegerin expression in human colorectal cancer. *Clinical Cancer Research*.

[44] Matsuyama T., Ishikawa T., Mogushi K. (2010). MUC12 mRNA expression is an independent marker of prognosis in stage II and stage III colorectal cancer. *International Journal of Cancer*.

[45] Cristescu R., Lee J., Nebozhyn M. (2015). Molecular analysis of gastric cancer identifies subtypes associated with distinct clinical outcomes. *Nature Medicine*.

[46] Sano T., Coit D. G., Kim H. H. (2017). Proposal of a new stage grouping of gastric cancer for TNM classification: International Gastric Cancer Association staging project. *Gastric Cancer*.

[47] Pohlodek K., Tan Y. Y., Singer C. F., Gschwantler-Kaulich D. (2016). Cadherin-11 expression is upregulated in invasive human breast cancer. *Oncology Letters*.

[48] Satriyo P., Bamodu O., Chen J.-H. (2019). Cadherin 11 inhibition downregulates *β*-catenin, deactivates the canonical WNT signalling pathway and suppresses the cancer stem cell-like phenotype of triple negative breast cancer. *Journal of Clinical Medicine*.

[49] Chen P. F., Wang F., Nie J. Y. (2018). Co-expression network analysis identified CDH11 in association with progression and prognosis in gastric cancer. *OncoTargets and Therapy*.

[50] Lahens N. F., Ricciotti E., Smirnova O. (2017). A comparison of Illumina and Ion Torrent sequencing platforms in the context of differential gene expression. *BMC Genomics*.

[51] Rhee J. K., Jung Y. C., Kim K. R. (2018). Impact of tumor purity on immune gene expression and clustering analyses across multiple cancer types. *Cancer Immunology Research*.

[52] Kim N. H., Choi S. H., Lee T. R., Lee C. H., Lee A. Y. (2014). Cadherin 11, a miR-675 target, induces N-cadherin expression and epithelial-mesenchymal transition in melasma. *The Journal of Investigative Dermatology*.

[53] Yao J., Deng B., Zheng L., Dou L., Guo Y., Guo K. (2016). miR-27b is upregulated in cervical carcinogenesis and promotes cell growth and invasion by regulating CDH11 and epithelial-mesenchymal transition. *Oncology Reports*.

[54] Shen Y., Song J., Wang Y. (2019). M2 macrophages promote pulmonary endothelial cells regeneration in sepsis-induced acute lung injury. *Annals of Translational Medicine*.

[55] Santander A., Lopez-Ocejo O., Casas O. (2015). Paracrine interactions between adipocytes and tumor cells recruit and modify macrophages to the mammary tumor microenvironment: the role of obesity and inflammation in breast adipose tissue. *Cancers*.

[56] Chatterjee M., von Ungern-Sternberg S. N. I., Seizer P. (2015). Platelet-derived CXCL12 regulates monocyte function, survival, differentiation into macrophages and foam cells through differential involvement of CXCR4-CXCR7. *Cell Death & Disease*.

[57] Zhou H., Wu J., Wang T., Zhang X., Liu D. (2016). CXCL10/CXCR3 axis promotes the invasion of gastric cancer via PI3K/AKT pathway-dependent MMPs production. *Biomedicine & pharmacotherapy*.

[58] Fanelli M. F., Chinen L. T. D., Begnami M. D. (2012). The influence of transforming growth factor-*α*, cyclooxygenase-2, matrix metalloproteinase (MMP)-7, MMP-9 and CXCR4 proteins involved in epithelial-mesenchymal transition on overall survival of patients with gastric cancer. *Histopathology*.

[59] Yao Z., Zhang J., Zhang B. (2018). Imatinib prevents lung cancer metastasis by inhibiting M2-like polarization of macrophages. *Pharmacological Research*.

[60] Vu T., Datta P. K. (2017). Regulation of EMT in colorectal cancer: a culprit in metastasis. *Cancers*.

[61] Brown N. F., Marshall J. F. (2019). Integrin-mediated TGF*β* activation modulates the tumour microenvironment. *Cancers*.

[62] Markiewicz A., Topa J., Nagel A. (2019). Spectrum of epithelial-mesenchymal transition phenotypes in circulating tumour cells from early breast cancer patients. *Cancers*.

[63] Brown G. T., Murray G. I. (2015). Current mechanistic insights into the roles of matrix metalloproteinases in tumour invasion and metastasis. *The Journal of Pathology*.

[64] Singh D., Srivastava S. K., Chaudhuri T. K., Upadhyay G. (2015). Multifaceted role of matrix metalloproteinases (MMPs). *Frontiers in Molecular Biosciences*.

[65] Liu H., Zhang W., Wang L. (2019). GLI1 is increased in ovarian endometriosis and regulates migration, invasion and proliferation of human endometrial stromal cells in endometriosis. *Annals of Translational Medicine*.

[66] Lamouille S., Xu J., Derynck R. (2014). Molecular mechanisms of epithelial-mesenchymal transition. *Nature Reviews. Molecular Cell Biology*.

