# Supplementary material for: Prognostic and Predictive Value of Cadherin 11 for Patients with Gastric Cancer and Its Correlation with Tumor Microenvironment: Results from Microarray Analysis
Source: Biomed Res Int. 2020 Jun 26;2020:8107478. doi: 10.1155/2020/8107478 (PMC7335407; doi:10.1155/2020/8107478)
Supplement: Supplementary Materials — Figure S1: expression levels of CDH11 in various human cancers from the GEPIA database. Figure S2: Kaplan-Meier survival curves comparing the high and low expressions of CDH11 in various cancers from the GEPIA database. Figure S3: different levels of CDH11 expression between different lymph node metastases of GC patients. Table S1: CDH11 expression in gastric, colorectal, and pancreatic cancers from the Oncomine database. Table S2: the information of datasets used for differential analysis in the study. Table S3: the relationship between CDH11 and disease progression in patients with gastric cancer. [file 8107478.f1.zip › Supplementary files/Table S1 CDH11 expression in gastric, colorectal and pancreaticcancers from the Oncomine database.docx]

| **Datasets** | **No.** | **Midia(Quartiles)** | **FC** | **T** | ***P*-Value** |
| --- | --- | --- | --- | --- | --- |
| **Chen Gastric Statistics** |  |  |  |  |  |
| Gastric Mucosa | 29 | 0.223(0.048-0.617) | 2.219 | 11.152 | 9.46E-18 |
| Gastric Intestinal Type Adenocarcinoma | 63 | 1.393(1.091-1.889) |  |  |  |
| Gastric Mucosa | 29 | 1.765(1.693-1.864) | 2.200 | 6.953 | 6.86E-07 |
| Diffuse Gastric Adenocarcinoma | 13 | 1.632(1.126-1.894) |  |  |  |
| Gastric Mucosa | 29 | 1.765(1.693-1.864) | 4.087 | 9.236 | 1.01E-05 |
| Gastric Mixed Adenocarcinoma | 7 | 2.494(1.873-3.176) |  |  |  |
| **DErrico Gastric Statistics** |  |  |  |  |  |
| Gastric Mucosa | 31 | 2.881(2.558-3.233) | 4.962 | 15.205 | 2.50E-09 |
| Gastric Mixed Adenocarcinoma | 4 | 5.034(4.874-5.239) |  |  |  |
| Gastric Mucosa | 31 | 2.881(2.558-3.233) | 2.773 | 5.888 | 4.64E-07 |
| Gastric Intestinal Type Adenocarcinoma | 26 | 4.256(3.209-5.149) |  |  |  |
| **Wang Gastric Statistics** |  |  |  |  |  |
| Gastric Mucosa | 12 | 3.135(2.938-3.216) | 3.284 | 6.512 | 1.10E-06 |
| Gastric Tissue | 3 | 2.681(2.406-2.803) |  |  |  |
| Gastric Cancer | 12 | 4.748(4.564-5.485) |  |  |  |
| **Cui Gastric Statistics** |  |  |  |  |  |
| Gastric Tissue | 80 | 0.594(0.245-1.204) | 2.042 | 6.799 | 1.17E-10 |
| Gastric Cancer | 80 | 1.884(1.064-2.537) |  |  |  |
| **Cho Gastric Statistics** |  |  |  |  |  |
| Gastric Tissue | 19 | 3.461(3.125-3.667) | 2.540 | 6.929 | 4.79E-09 |
| Diffuse Gastric Adenocarcinoma | 31 | 4.781(4.215-5.451) |  |  |  |
| **Kaiser Colon Statistics** |  |  |  |  |  |
| Colon | 5 | 1.765(1.693-1.864) | 3.022 | 13.112 | 4.28E-17 |
| Colon Adenocarcinoma | 41 | 3.421(2.827-3.891) |  |  |  |
| Colon | 5 | 1.765(1.693-1.864) | 4.934 | 10.075 | 1.03E-07 |
| Colon Mucinous Adenocarcinoma | 13 | 4.433(3.436-4.595) |  |  |  |
| Colon | 5 | 1.765(1.693-1.864) | 2.736 | 6.581 | 2.38E-06 |
| Cecum Adenocarcinoma | 17 | 3.098(2.438-3.623) |  |  |  |
| Colon | 5 | 1.765(1.693-1.864) | 3.388 | 6.181 | 6.99E-05 |
| Rectosigmoid Adenocarcinoma | 10 | 3.79(3.167-4.057) |  |  |  |
| **Skrzypczak Colorectal 2 Statistics** |  |  |  |  |  |
| Colon | 10 | 1.157(0.965-1.418) | 5.758 | 15.748 | 7.14E-10 |
| Colon Carcinoma | 5 | 3.714(3.380-3.868) |  |  |  |
| Colon | 10 | 3.730(3.521-4.013) | 3.381 | 16.976 | 1.31E-09 |
| Colon Carcinoma | 5 | 5.445(5.418-5.606) |  |  |  |
| **Skrzypczak Colorectal Statistics** |  |  |  |  |  |
| Colorectal Tissue | 24 | 3.068(2.845-3.392) | 3.008 | 6.726 | 4.72E-09 |
| Colorectal Carcinoma | 36 | 4.926(4.383-5.559) |  |  |  |
| **Badea Pancreas Statistics** |  |  |  |  |  |
| Pancreas | 39 | 0.384(0.069-1.842) | 5.276 | 10.517 | 2.23E-15 |
| Pancreatic Ductal Adenocarcinoma | 39 | 3.363(2.698-3.695) |  |  |  |
| **Pei Pancreas Statistics** |  |  |  |  |  |
| Pancreas | 16 | 4.905(2.865-7.096) | 3.98 | 2.999 | 4.00E-03 |
| Pancreatic Carcinoma | 36 | 7.088(6.254-7.413) |  |  |  |

**Table S1** CDH11 expression in gastric, colorectal and pancreatic cancers from the Oncomine database. No., the samples number of corresponding database; FC, the value of fold change; T, the value of the statistical test.
