# Supplementary material for: Prognostic and Predictive Value of Cadherin 11 for Patients with Gastric Cancer and Its Correlation with Tumor Microenvironment: Results from Microarray Analysis
Source: Biomed Res Int. 2020 Jun 26;2020:8107478. doi: 10.1155/2020/8107478 (PMC7335407; doi:10.1155/2020/8107478)
Supplement: Supplementary Materials — Figure S1: expression levels of CDH11 in various human cancers from the GEPIA database. Figure S2: Kaplan-Meier survival curves comparing the high and low expressions of CDH11 in various cancers from the GEPIA database. Figure S3: different levels of CDH11 expression between different lymph node metastases of GC patients. Table S1: CDH11 expression in gastric, colorectal, and pancreatic cancers from the Oncomine database. Table S2: the information of datasets used for differential analysis in the study. Table S3: the relationship between CDH11 and disease progression in patients with gastric cancer. [file 8107478.f1.zip › Supplementary files/Table S2 The information of datasets used for differential analysis in the study.docx]

| **Series** | **Types** | **No. of T** | **No. of N** | **Platform** | **Country** |
| --- | --- | --- | --- | --- | --- |
| GSE66229 | STAD | 300 | 100 | GPL570 | USA |
| GSE54129 | STAD | 111 | 21 | GPL570 | China |
| GSE13911 | STAD | 38 | 31 | GPL570 | Italy |
| GSE15471 | PAAD | 39 | 39 | GPL570 | Romania |
| GSE16515 | PAAD | 36 | 16 | GPL570 | USA |
| GSE21510 | COLO | 123 | 25 | GPL570 | Japan |
| GSE18105 | COLO | 94 | 17 | GPL570 | Japan |

**Table S2** The information of datasets used for differential analysis in the study. No. of T, the sample number of tumor tissues; No. of N, the sample number of normal tissues. STAD, stomach adenocarcinoma; PAAD, pancreatic adenocarcinoma; COLO, colorectal cancer.
