# Supplementary material for: Prognostic and Predictive Value of Cadherin 11 for Patients with Gastric Cancer and Its Correlation with Tumor Microenvironment: Results from Microarray Analysis
Source: Biomed Res Int. 2020 Jun 26;2020:8107478. doi: 10.1155/2020/8107478 (PMC7335407; doi:10.1155/2020/8107478)
Supplement: Supplementary Materials — Figure S1: expression levels of CDH11 in various human cancers from the GEPIA database. Figure S2: Kaplan-Meier survival curves comparing the high and low expressions of CDH11 in various cancers from the GEPIA database. Figure S3: different levels of CDH11 expression between different lymph node metastases of GC patients. Table S1: CDH11 expression in gastric, colorectal, and pancreatic cancers from the Oncomine database. Table S2: the information of datasets used for differential analysis in the study. Table S3: the relationship between CDH11 and disease progression in patients with gastric cancer. [file 8107478.f1.zip › Supplementary files/Table S3 The relationship between CDH11 and disease progression in patients with gastric cancer.docx]

| **Evaluation category** | **Log rank p** | **Hazard ratio (HR)** | **95% Confidence interval (CI)** | **Data Sources** |
| --- | --- | --- | --- | --- |
| DFS | 4.20E-01 | 1.20 | NA | GEPIA database |
| RFS | 2.80E-02 | 2.57 | 1.07-6.07 | RNA-seq data in KM-plotter |
| FP (207172_s_at) | 2.60E-06 | 1.63 | 1.33-2.00 | Gene-chip data in KM-plotter |
| PPS (207172_s_at) | 7.00E-08 | 1.70 | 1.40-2.06 | Gene-chip data in KM-plotter |
| FP (207173_x_at) | 3.20E-04 | 1.48 | 1.19-1.83 | Gene-chip data in KM-plotter |
| PPS (207173_x_at) | 1.10E-05 | 1.68 | 1.33-2.12 | Gene-chip data in KM-plotter |
| FP (236179_at) | 4.50E-10 | 2.09 | 1.65-2.65 | Gene-chip data in KM-plotter |
| PPS (236179_at) | 3.70E-08 | 2.11 | 1.61-2.77 | Gene-chip data in KM-plotter |
| DFS | 1.00E-03 | 2.60 | 1.50-4.80 | GSE62254 |
| RFS | 4.00E-03 | 1.20 | 1.10-1.40 | GSE26253 |

Table S3 The relationship between CDH11 and disease progression in patients with gastric cancer. DFS, disease-free survival; RFS, recurrence-free survival; FP, first progression; PPS, post-progression survival; NA, not available; GEPIA, Gene Expression Profiling Interactive Analysis.
