# Supplementary material for: Prognostic and Predictive Value of Cadherin 11 for Patients with Gastric Cancer and Its Correlation with Tumor Microenvironment: Results from Microarray Analysis
Source: Biomed Res Int. 2020 Jun 26;2020:8107478. doi: 10.1155/2020/8107478 (PMC7335407; doi:10.1155/2020/8107478)
Supplement: Supplementary Materials — Figure S1: expression levels of CDH11 in various human cancers from the GEPIA database. Figure S2: Kaplan-Meier survival curves comparing the high and low expressions of CDH11 in various cancers from the GEPIA database. Figure S3: different levels of CDH11 expression between different lymph node metastases of GC patients. Table S1: CDH11 expression in gastric, colorectal, and pancreatic cancers from the Oncomine database. Table S2: the information of datasets used for differential analysis in the study. Table S3: the relationship between CDH11 and disease progression in patients with gastric cancer. [file 8107478.f1.zip › Supplementary files/The description of Supplementary FigureS1-3.docx]

**Figures**

**Figure S1** Expression levels of CDH11 in various human cancers from GEPIA database. (a - q) Expression levels of CDH11 in BLCA (Bladder Urothelial Carcinoma), BRCA (Breast invasive carcinoma), CHOL (Cholangiocarcinoma), COAD (Colon adenocarcinoma), HNSC (Head and Neck squamous cell carcinoma), KICH (Kidney Chromophobe), KIRC (Kidney renal clear cell carcinoma), KIRP (Kidney renal papillary cell carcinoma), LIHC (Liver hepatocellular carcinoma), LUSC (Lung squamous cell carcinoma), PAAD (Pancreatic adenocarcinoma), PRAD (Prostate adenocarcinoma), READ (Rectum adenocarcinoma), SARC (Sarcoma), STAD (Stomach adenocarcinoma), THCA (Thyroid carcinoma), and UCEC (Uterine Corpus Endometrial Carcinoma). Red represents expression levels in tumor tissues and gray represents expression levels in normal tissues. * p-value < 0.05.

**Figure S2** Kaplan-Meier survival curves comparing the high and low expression of CDH11 in various cancers from GEPIA database. (a-j) Survival curves of overall survival (OS) in BLCA (Bladder Urothelial Carcinoma), BRCA (Breast invasive carcinoma), ESCA (Esophageal carcinoma), HNSC (Head and Neck squamous cell carcinoma), KIRC (Kidney renal clear cell carcinoma), KIRP (Kidney renal papillary cell carcinoma), LIHC (Liver hepatocellular carcinoma), LUAD (Lung adenocarcinoma), LUSC (Lung squamous cell carcinoma), and SARC (Sarcoma). HR, hazard ratio.

**Figure S3** Different levels of CDH11 expression between different lymph node metastasis of GC patients. (A) CDH11 expression in N1-3 (lymph node metastasis) compared with N0 (no lymph node metastasis) in GSE62254 from GEO database. (B) CDH11 expression in N0 (no lymph node metastasis), N1 (Number of lymph node metastasis region less than 2), N2 (Number of lymph node metastasis region between 3 and 6), N3 (Number of lymph node metastasis region greater than or equal to 7) in GSE84437 from GEO database.
