# Supplementary figures and images for: Prognostic and Predictive Value of Cadherin 11 for Patients with Gastric Cancer and Its Correlation with Tumor Microenvironment: Results from Microarray Analysis
Source: Biomed Res Int. 2020 Jun 26;2020:8107478. doi: 10.1155/2020/8107478 (PMC7335407; doi:10.1155/2020/8107478)

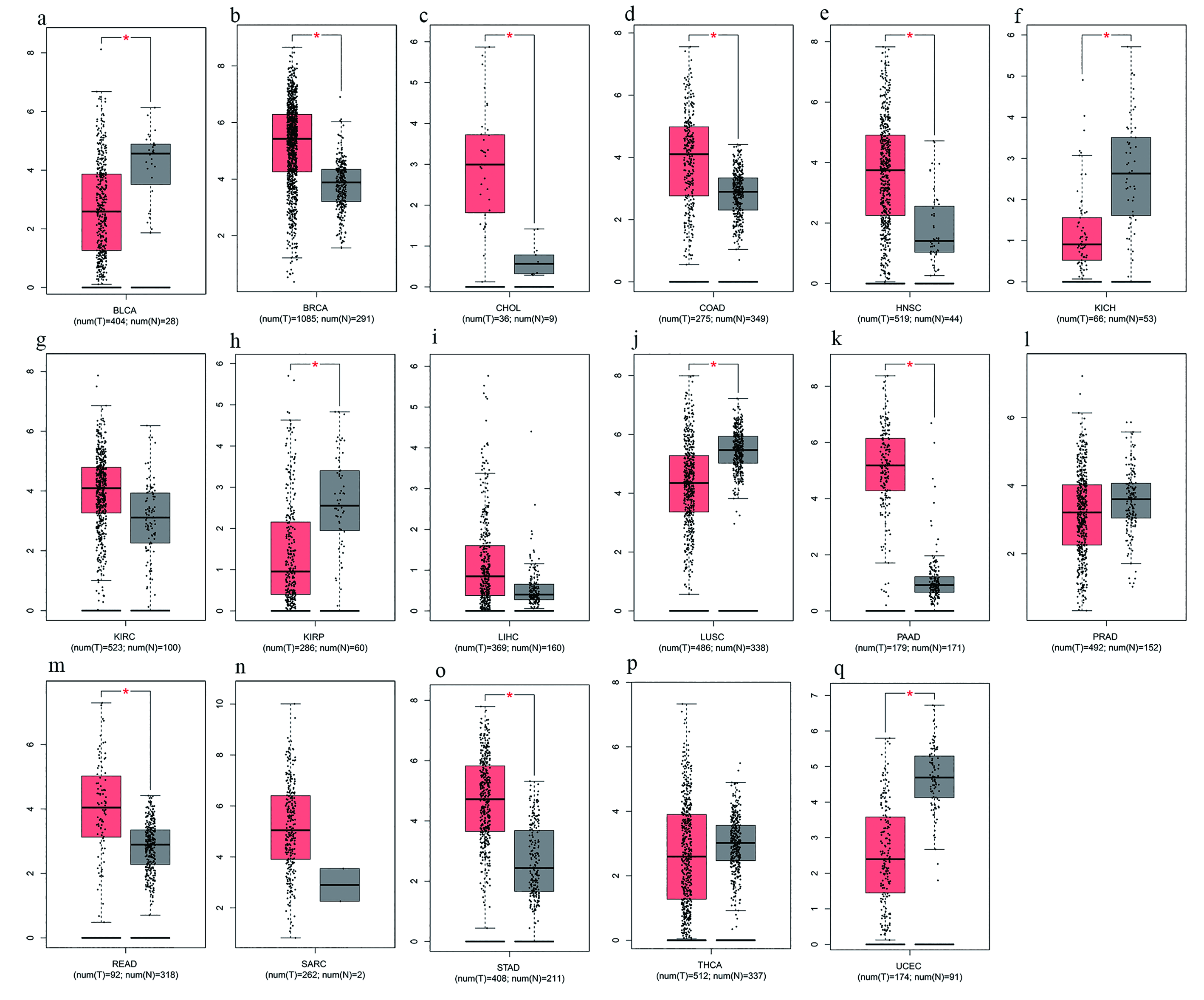

Supplement: Supplementary Materials — Figure S1: expression levels of CDH11 in various human cancers from the GEPIA database. Figure S2: Kaplan-Meier survival curves comparing the high and low expressions of CDH11 in various cancers from the GEPIA database. Figure S3: different levels of CDH11 expression between different lymph node metastases of GC patients. Table S1: CDH11 expression in gastric, colorectal, and pancreatic cancers from the Oncomine database. Table S2: the information of datasets used for differential analysis in the study. Table S3: the relationship between CDH11 and disease progression in patients with gastric cancer. [file 8107478.f1.zip › Supplementary files/Figure S1 Expression levels of CDH11 in various human cancers in the GEPIA database.tif]

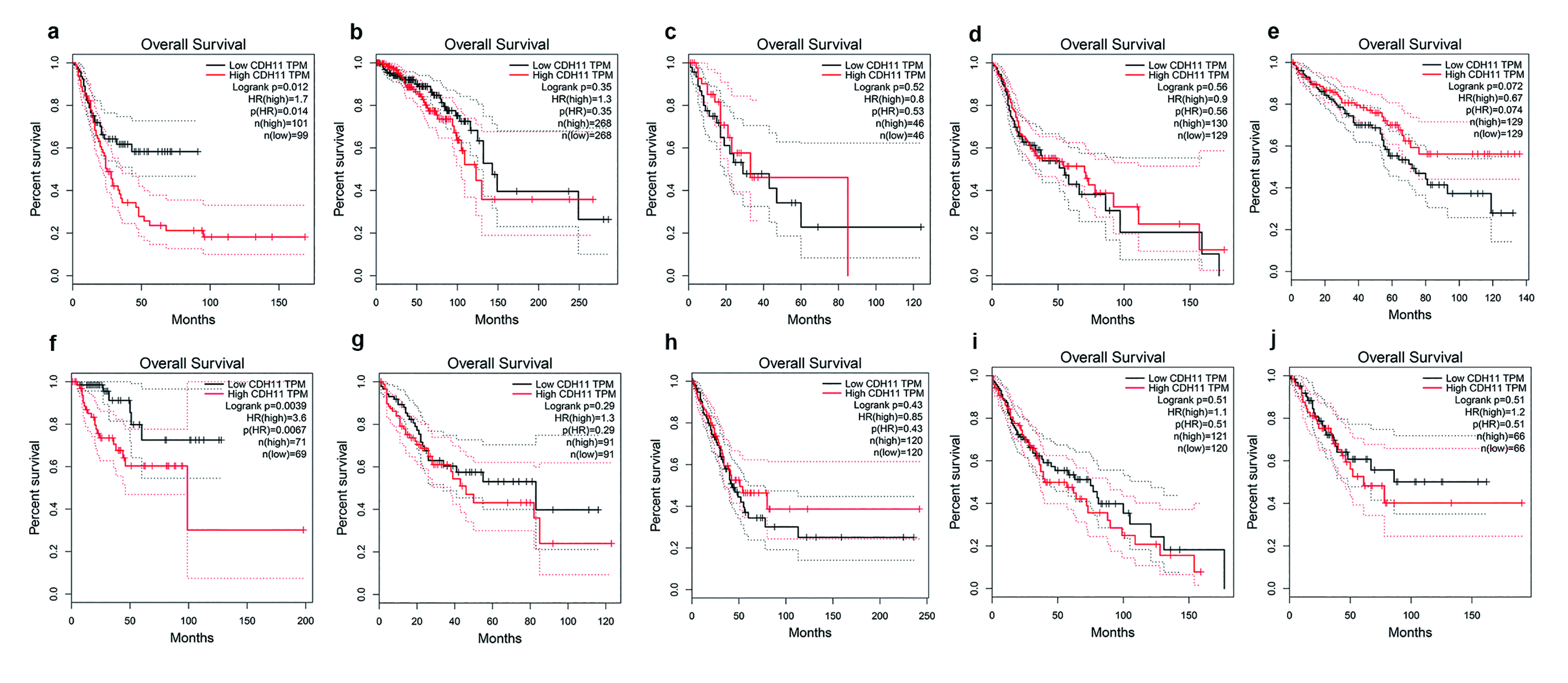

Supplement: Supplementary Materials — Figure S1: expression levels of CDH11 in various human cancers from the GEPIA database. Figure S2: Kaplan-Meier survival curves comparing the high and low expressions of CDH11 in various cancers from the GEPIA database. Figure S3: different levels of CDH11 expression between different lymph node metastases of GC patients. Table S1: CDH11 expression in gastric, colorectal, and pancreatic cancers from the Oncomine database. Table S2: the information of datasets used for differential analysis in the study. Table S3: the relationship between CDH11 and disease progression in patients with gastric cancer. [file 8107478.f1.zip › Supplementary files/f2.tif]

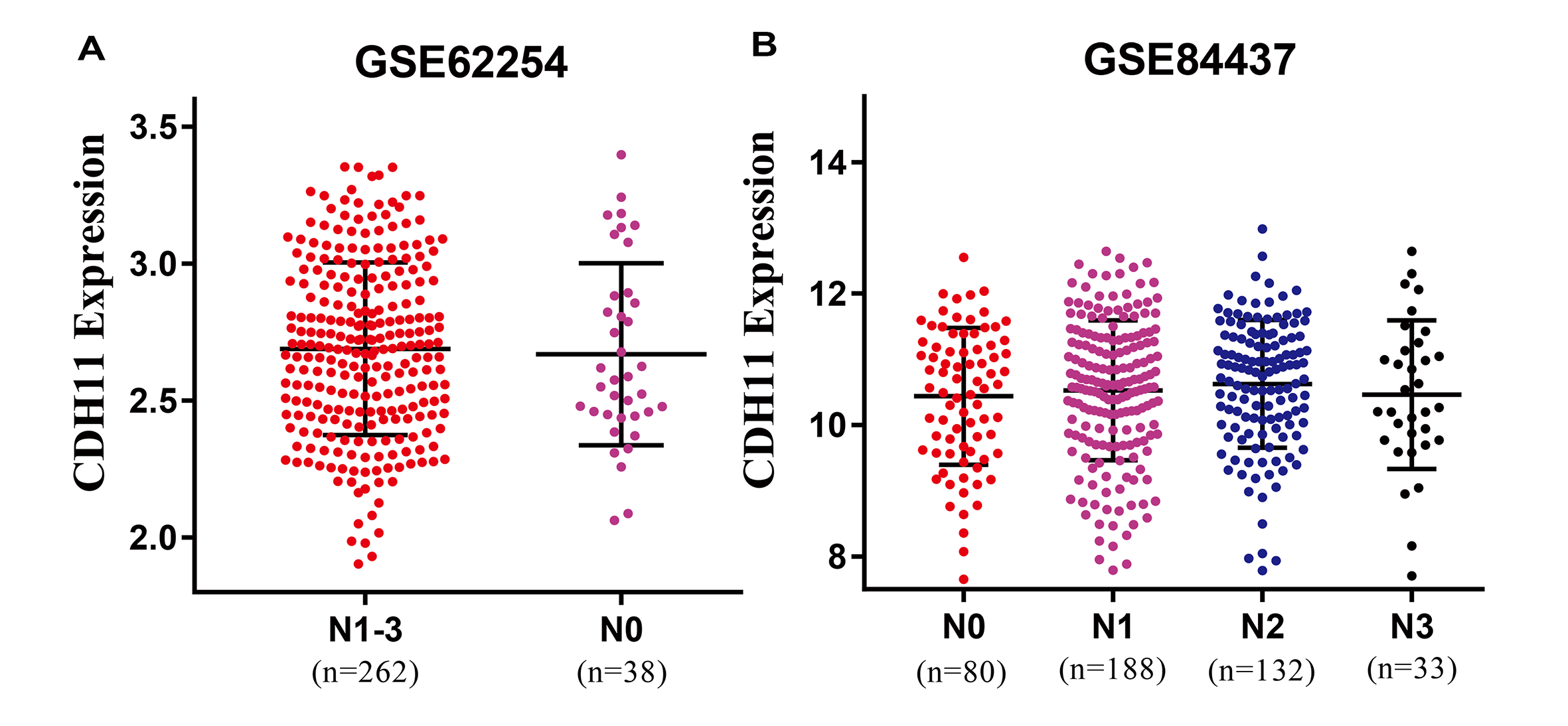

Supplement: Supplementary Materials — Figure S1: expression levels of CDH11 in various human cancers from the GEPIA database. Figure S2: Kaplan-Meier survival curves comparing the high and low expressions of CDH11 in various cancers from the GEPIA database. Figure S3: different levels of CDH11 expression between different lymph node metastases of GC patients. Table S1: CDH11 expression in gastric, colorectal, and pancreatic cancers from the Oncomine database. Table S2: the information of datasets used for differential analysis in the study. Table S3: the relationship between CDH11 and disease progression in patients with gastric cancer. [file 8107478.f1.zip › Supplementary files/Figure S3 Expression levels of CDH11 in gastric cancer with lymph node metastasis in the GEO database.tif]
